# Comparison of systemic conditions at diagnosis between central retinal vein occlusion and branch retinal vein occlusion

**DOI:** 10.1371/journal.pone.0220880

**Published:** 2019-08-08

**Authors:** Bum-Joo Cho, So Hyun Bae, Sang Min Park, Min Chul Shin, In Won Park, Ha Kyoung Kim, Soonil Kwon

**Affiliations:** 1 Department of Ophthalmology, Hallym University College of Medicine, Hallym University Sacred Heart Hospital, Anyang, Korea; 2 Department of Ophthalmology, Hallym University College of Medicine, Kangnam Sacred Heart Hospital, Seoul, Korea; 3 Cardiovascular Center, Hallym University College of Medicine, Chuncheon Sacred Heart Hospital, Chuncheon, Korea; 4 Department of Ophthalmology, Hallym University College of Medicine, Chuncheon Sacred Heart Hospital, Chuncheon, Korea; University of Melbourne, AUSTRALIA

## Abstract

**Objective:**

To compare systemic conditions at the time of diagnosis between patients with central retinal vein occlusion (CRVO) and branch retinal vein occlusion (BRVO).

**Design:**

This study included patients diagnosed with CRVO or BRVO between February 2009 and August 2017 at three branch hospitals of Hallym University Medical Center. Demographic and anthropometric variables, systemic comorbidity profiles, and laboratory findings at diagnosis were collected from a clinical data warehouse system, and were compared between the CRVO and BRVO groups.

**Result:**

Four hundred and seventeen patients with CRVO and 1,511 patients with BRVO were included. The mean age was 61.8 ± 13.9 years, which was comparable between two groups (*P* = .332). Female proportion was higher in the BRVO group (55.0%) than in the CRVO group (48.0%; *P* = .013). Diabetes mellitus (*P* = .017) and chronic kidney disease (*P* = .004) were more prevalent in the CRVO group. Serum homocysteine level was abnormally high in 23.5% of CRVO patients and in 8.4% of BRVO patients (*P* < .001). Blood urea nitrogen and serum creatinine levels were abnormally elevated in more subjects with CRVO (*P* = .002).

**Conclusion:**

CRVO is associated with higher prevalence of diabetes mellitus and chronic kidney disease, as well as with elevated serum homocysteine level. These results might suggest a difference between the pathophysiologies of CRVO and BRVO.

## Introduction

Retinal vein occlusion (RVO) is the most common retinal vascular disease after diabetic retinopathy, and it is one of the major causes of visual impairment worldwide [1, 2]. It is categorized as central RVO (CRVO) and branch RVO (BRVO) depending on the site of closure, and these two subtypes differ with regard to the clinical course and visual prognosis [2, 3]. Of note, the pathogenesis of RVO is still not completely understood, and it remains unclear whether the mechanisms underlying CRVO and BRVO are similar [4].

To reveal the pathophysiology of different types of RVO, investigation of systemic conditions combined with RVO may provide useful clues. Thus far, the development of RVO has been associated with obesity, smoking, and hemorheological factors [4–6]. Systemic conditions have been also reported as risk factors, such as hypertension, diabetes, dyslipidemia, and other cardiovascular diseases [7]. Notably, BRVO was associated with a higher prevalence of arterial hypertension, peripheral vascular disease, and atherosclerosis, compared to CRVO [8–10]. Genetic profile and some laboratory test results such as uric acid, glucose, and anti-nuclear antibody were also different between patients with BRVO and those with CRVO [11, 12]. Nevertheless, direct comparison on both systemic comorbidities and comprehensive laboratory test results between BRVO and CRVO patients in the same institution is limited yet, especially for Asian ethnics.

In this study, we aimed to investigate differences in systemic conditions including various laboratory results at diagnosis between CRVO and BRVO. To explore the large database of three hospitals collecting clinical information of patients with each type of RVO, we used the common integrated clinical data warehouse (CDW) system of the hospitals, which is an electronic data repository of patient and provider information [13]. This is one of the largest study on the association of systemic diseases with BRVO and CRVO in Asians. The findings of this study are expected to provide insight into the differences in the pathogenesis of CRVO and BRVO.

## Materials and methods

### Study population

The dataset analyzed in the current study was acquired from the common integrated electronic CDW system of Hallym University Medical Center (HUMC) [14]. The common CDW system of HUMC collects and stores extensive electronic medical data including medical records, laboratory results, physical measurements, diagnostic and therapeutic history, and medication history, over a period of 10 years from the five branch hospitals of HUMC [14]. We accessed the CDW system and investigated the medical data of patients who were newly diagnosed with RVO between February 2009 and August 2017 at any of three branch hospitals of HUMC: Hallym University Sacred Heart Hospital (HSHH), Kangnam Sacred Heart Hospital (KSHH), and Chuncheon Sacred Heart Hospital (CSHH). This study was approved by the institutional review board of HUMC, and all protocols were in accordance with the tenets of the Declaration of Helsinki. Informed consent was waived because of the retrospective nature of the study and the de-identification by the CDW system before we access the database.

We first identified patients diagnosed with RVO [Korean Standard Classification of Diseases (KCD) code H34.8, corresponding to the International Classification of Diseases, 9^th^ Revision, Clinical Modification (ICD-9-CM) code 362.35 for CRVO or 362.36 for BRVO) during the period mentioned above. If a patient developed both types of RVO during the clinical course, the initially occurring type was included. Next, to ensure the inclusion of patients with new episodes only, we verified the presence of a previous RVO diagnosis by reviewing the visit data for all eligible patients, beginning from the earliest period for which medical records were provided by the CDW system (January 2007 for HSHH, February 2008 for KSHH, and March 2007 for CSHH). We evaluated data pertaining to all visits made during ≥ 1 year before RVO diagnosis. All patients with a previous diagnosis of RVO were then excluded.

### Main outcome measures

The data of study subjects were investigated with regard to the systemic conditions at the time of RVO diagnosis. The electronic CDW system of HUMC was used to derive data pertaining to the demographic characteristics of patients, underlying systemic comorbidities, physical measurements, and laboratory findings of blood tests and urine tests. For anthropometric values and laboratory findings, test results from 14 days before to 7 days after the diagnosis of RVO were selectively included for analysis. When two or more test results were available, values obtained at the date closest to the date of RVO diagnosis were selected. Systemic diseases diagnosed before RVO diagnosis were defined as underlying comorbidities.

Demographic characteristics included the patient’s sex and age at RVO diagnosis. Systemic comorbidities were investigated using the KCD code system. The prevalence rates of the 10 most common diseases in our RVO patients, including diabetes mellitus (DM), hypertension, ischemic heart disease (IHD), dyslipidemia, cerebral infarction, cerebral hemorrhage, arrhythmia, chronic kidney disease diseases (CKD), gastroduodenal ulcer or inflammation, and benign prostatic hyperplasia were compared between the two groups. The specific criteria for the diagnosis of systemic diseases are presented in S1 Table.

Physical measurements included height, weight, systolic blood pressure (SBP), diastolic blood pressure (DBP), and the body mass index (BMI). The laboratory protocol for RVO included a complete blood count with differential [white blood cells, hemoglobin, hematocrit, platelet count, etc.]; blood coagulation- related test [activated partial thromboplastin time, prothrombin time (PT), and serum homocysteine]; a lipid profile [total cholesterol, low-density lipoprotein cholesterol, high-density lipoprotein cholesterol, and triglycerides]; a liver function test, including alanine transaminase and aspartate aminotransferase (AST) levels; blood urea nitrogen (BUN) measurement, and creatinine measurement. Because some laboratory test results were unobtainable for some patients, the tests of which results were available in more than 10% of the whole study participants were included in the analyses. The absolute values of laboratory findings and the proportion of subjects with abnormal findings were compared between the two groups.

### Statistical analyses

The patients were assigned to a BRVO group and a CRVO group. Then, the outcome variables were compared between the groups. Continuous variables were expressed as means ± standard deviations, and categorical variables were expressed as the number and proportion of patients. Chi-square tests, Fisher’s exact tests, independent t-tests, and paired t-tests were used for statistical analyses. A *P*-value of < .05 was considered statistically significant. All statistical analyses were performed using R version 3.3.3 (The R Foundation for Statistical Computing, Vienna, Austria).

### Data availability

Data supporting the findings of the current study are available from the corresponding author on reasonable request.

## Results

In total, 1,928 patients with RVO were included: 753 patients were recruited from HSHH, 595 patients were from KSHH, and 580 patients were from CSHH. There was no difference in age, the proportion of patients with CRVO, the proportion of male patients, and the BMI (*P* = .468, *P* = .424, *P* = .211, and *P* = .645, respectively) among the three hospitals. In the entire group, mean age at diagnosis was 61.8 ± 13.9 years and 897 (46.5%) subjects were male.

Among the entire group, 417 and 1,511 patients were diagnosed with CRVO and BRVO, respectively. The demographic characteristics of the patients are presented in Table 1. The mean age was 61.2 ± 16.7 years in the CRVO group and 62.0 ± 13.1 years in the BRVO group (*P* = .332). The BRVO group had a significantly higher proportion of female patients than did the CRVO group (55.0% vs. 48.0%, respectively; *P* = .013). DBP was significantly lower in patients with CRVO than in patients with BRVO (77.4 ± 16.8 mmHg vs. 84.5 ± 17.1 mmHg, respectively; *P* = .011). There were no significant differences in the other variables, including height, weight, BMI, and SBP (*P* > .05 for all).

**Table 1:** Demographic characteristics of patients with branch retinal vein occlusion and those with central retinal vein occlusion.

	CRVO		BRVO		*P*-value
	N	Values	N	Values	
**Age (years)**	417	61.2 ± 16.7	1511	62.0 ± 13.1	.332^**a**^
**Sex, Male (%)**	417	217 (52.0%)	1511	680 (45.0%)	.013^**b**^
**Height (cm)**	44	160.8 ± 8.6	61	160.6 ± 9.1	.879^**a**^
**Weight (Kg)**	48	62.4 ± 11.2	76	64.7 ± 11.5	.293^**a**^
**BMI (Kg/m^2^)**	44	23.8 ± 2.9	61	24.8 ± 3.5	.124^**a**^
**SBP (mmHg)**	56	131.4 ± 29.0	130	138.0 ± 27.8	.140^**a**^
**DBP (mmHg)**	56	77.4 ± 16.8	130	84.5 ± 17.1	.011^**a**^

CRVO: central retinal vein occlusion, BRVO: branch retinal vein occlusion, BMI: body mass index, SBP: systolic blood pressure, DBP: diastolic blood pressure

^**a**^Independent t-test

^**b**^Pearson’s chi-square test

### Systemic comorbidities

Table 2 presents the systemic disease profile at the time of RVO diagnosis. Hypertension was the most common underlying disease (17.3% in CRVO and 15.2% in BRVO), followed by DM. The CRVO group showed a higher prevalence of DM and CKD than did the BRVO group (10.1% vs. 6.5% for DM, *P* = .017; 4.3% vs. 1.8% for CKD, *P* = .004). There was no significant difference in the prevalence rates of other comorbid diseases between the two groups.

**Table 2:** Systemic comorbidities in patients with branch retinal vein occlusion and those with central retinal vein occlusion.

	CRVO (N = 417)	BRVO (N = 1511)	*P*-value
**Hypertension**	72 (17.3%)	229 (15.2%)	.330^**a**^
**Diabetes mellitus**	42 (10.1%)	98 (6.5%)	.017^**a**^
**Ischemic heart disease**	25 (6.0%)	63 (4.2%)	.147^**a**^
**Dyslipidemia**	22 (5.3%)	85 (5.6%)	.877^**a**^
**Cerebral infarction**	10 (2.4%)	53 (3.5%)	.331^**a**^
**Cerebral hemorrhage**	2 (0.5%)	10 (0.7%)	0.946^**b**^
**Arrhythmia**	9 (2.2%)	38 (2.5%)	0.811^**a**^
**Chronic kidney disease**	18 (4.3%)	27 (1.8%)	.004^**a**^
**Gastroduodenal ulcer or inflammation**	32 (7.7%)	137 (9.1%)	.428^**a**^
**Benign prostatic hyperplasia**	18 (4.3%)	50 (3.3%)	.402^**a**^

BRVO: branch retinal vein occlusion, CRVO: central retinal vein occlusion

^**a**^Pearson’s chi-square test

^**b**^Fisher’s exact test

### Laboratory tests

Tables 3 and 4 show the laboratory findings of patients with BRVO and CRVO. The serum hemoglobin level was significantly lower in the CRVO group than in the BRVO group (13.3 g/dL vs. 13.7 g/dL, respectively; *P* = .025). The prothrombin time was also shorter in the CRVO group (*P* = .004). Other coagulation-related findings, including serum platelet count, were comparable between the groups (*P* > .05 for all).

**Table 3:** Laboratory findings for patients with branch retinal vein occlusion and those with central retinal vein occlusion.

	Reference values	CRVO		BRVO		*P*-value
		N	Values	N	Values	
**ESR**	0–26 mm/h	92	13.4 ± 12.9	332	14.1 ± 14.1	.642^**a**^
**Homocysteine**	4.4–16.2 umol/L	81	13.3 ± 6.9	320	11.0 ± 5.2	.008^**a**^
**PT**	11.5–14.0 s	129	12.2 ± 1.4	423	12.7 ± 2.3	.004^**a**^
**aPTT**	29.1–45.1 s	128	34.6 ± 5.2	415	34.9 ± 4.8	.534^**a**^
**BUN**	10–26 mg/dL	166	17.5 ± 9.5	507	16.4 ± 7.9	.176^**a**^
**Creatinine**	0.6–1.2 mg/dL	166	1.2 ± 1.5	508	1.0 ± 1.4	.124^**a**^
**Albumin**	3.8–5.3 g/dL	163	4.4 ± 0.4	498	4.4 ± 0.3	.158^**a**^
**AST**	8–38 IU/L	166	22.4 ± 9.2	508	25.7 ± 15.0	.001^**a**^
**ALT**	5–43 IU/L	166	20.1 ± 13.4	507	22.6 ± 15.1	.056^**a**^
**Gamma GT**	11–75 IU/L	69	38.5 ± 44.0	151	40.7 ± 51.8	.769^**a**^
**Glucose**	70–110 mg/dL	158	128.0 ± 62.6	484	115.7 ± 43.6	0.023^**a**^
**Cholesterol**	130–200 mg/dL	163	188.2 ± 38.6	504	192.1 ± 39.9	0.276^**a**^
**TG**	40–150 mg/dL	106	141.8 ± 89.1	354	152.5 ± 91.8	0.290^**a**^
**LDL cholesterol**	0–130 mg/dL	103	116.2 ± 36.4	349	119.8 ± 33.6	0.345^**a**^
**HDL cholesterol**	>40 mg/dL	106	50.6 ±13.6	354	52.8 ± 13.0	0.125^**a**^

CRVO: central retinal vein occlusion, BRVO: branch retinal vein occlusion, ESR: erythrocyte sedimentation rate, PT: prothrombin time, aPTT: activated partial thromboplastin time, BUN: blood urea nitrogen, AST: aspartate aminotransferase, ALT: alanine aminotransferase, gamma GT: gamma-glutamyltransferase, TG: triglyceride, LDL: low-density lipoprotein, HDL: high-density lipoprotein

^**a**^Independent t-test

**Table 4:** Comparison of complete blood count results between patients with central retinal vein occlusion and those with branch retinal vein occlusion.

	Unit	CRVO		BRVO		*P*-value
		N	Values	N	Values	
**WBC**	10^3^/ul	164	6.5 ± 2.1	488	6.6 ± 1.8	.981^**a**^
**RBC**	10^6^/μL	164	4.4 ± 0.6	488	4.5 ± 0.5	.070^**a**^
**Hb**	g/dL	164	13.3 ± 2.0	489	13.7 ± 1.7	.025^**a**^
**Hct**	%	164	39.3 ± 5.4	487	40.4 ± 4.6	.026^**a**^
**Platelet**	10^3^/ul	164	241.7 ± 62.9	487	244.9 ±67.2	.595^**a**^
**Neutrophil**	10^9^/L	158	4.0 ± 1.8	471	3.8 ± 1.5	.158^**a**^
**Lymphocyte**	10^9^/L	80	1.8 ± 0.6	158	1.9 ± 0.7	.112^**a**^
**Neutrophil %**	%	163	59.3 ± 9.4	487	56.8 ± 9.6	.004^**a**^
**Lymphocyte %**	%	163	29.9 ± 8.0	487	32.2 ± 8.8	.003^**a**^
**Monocyte %**	%	163	6.0 ± 1.8	487	6.0 ± 1.9	.843^**a**^
**Eosinophil %**	%	163	2.6 ± 2.1	487	2.8 ± 2.7	.307^**a**^
**N/L ratio**		80	2.4 ± 1.2	158	2.4 ± 1.9	.942^**a**^
**N/L ratio ≥ 4.0**			8.8%		9.5%	1.000^**b**^

CRVO: central retinal vein occlusion, BRVO: branch retinal vein occlusion, WBC: white blood cell, RBC: red blood cell, Hb: hemoglobin, Hct: hematocrit, N/L ratio: neutrophil to lymphocyte ratio.

^**a**^Independent t-test

^**b**^Pearson’s chi-square test

The blood homocysteine level (13.3 ± 6.9 umol/L vs. 11.0 ± 5.2 umol/L, *P* = .008) was significantly higher in patients with CRVO than in those with BRVO. The proportion of patients with serum homocysteine level beyond the normal range was 23.5% in the CRVO group and 8.4% in the BRVO group (*P* < .001; Table 5). The serum glucose level was higher in the CRVO group than in the BRVO group (*P* = .023).

**Table 5:** The proportion of subjects with abnormal test results in patients with central retinal vein occlusion and those with branch retinal vein occlusion.

		CRVO	BRVO	*P*-value^a^
**Homocysteine**	>16.2 umol/L	23.5%	8.4%	.000
**PT**	>14 s	7.0%	5.2%	.583
**AST**	>38 IU/L	6.0%	7.9%	.536
**Glucose**	>110 mg/dL	41.8%	34.9%	.145
**BUN**	>26 mg/dL	12.0%	4.7%	.002
**Creatinine**	>1.2 mg/dL	13.3%	5.5%	.002

CRVO: central retinal vein occlusion, BRVO: branch retinal vein occlusion, PT: prothrombin time, AST: aspartate aminotransferase, BUN: blood urea nitrogen

^a^Chi-square test

Serum AST was significantly higher in patients with BRVO than in those with CRVO (*P* = .001), but these values were within the normal range in both groups. The serum cholesterol levels were not different between two groups (*P* > .05 for all). The CRVO group showed a higher proportion of neutrophils and a lower proportion of lymphocytes compared to the BRVO group (*P* = .004 and *P* = .003, respectively; Table 4). Other laboratory findings did not show any significant difference between the two groups (*P* > .05). Of note, patients with abnormally high level of serum BUN and creatinine were more prevalent in the CRVO group than in the BRVO group (*P* = .002 for both; Table 5).

Of note, 26 (6.2%) of 417 patients with CRVO developed BRVO during follow-up and 17 (1.1%) of 1,511 BRVO patients developed CRVO during follow-up within the study period. The ratio was significantly higher in patients who were firstly diagnosed with CRVO (*P* < .001). However, there were no significant difference between these two groups in terms of age (*P* = .103), sex (*P* = .994), anthropometric variables, and systemic comorbidities (*P* > .05 for all).

## Discussion

Risk factors for CRVO and BRVO have been reported by many studies, which have shown some overlap between the two diseases [15, 16]. Nevertheless, not many studies have compared systemic risk factors including laboratory findings for the two types of RVO. In the present study, we investigated differences in systemic conditions, including systemic comorbidities with consideration of laboratory findings, between CRVO and BRVO using a large dataset stored in an electronic CDW system. Compared with the BRVO group, the CRVO group showed male predominance, a higher prevalence of DM and CKD at the time of RVO diagnosis, elevated serum homocysteine levels, and a higher prevalence of abnormal serum BUN/creatinine levels. Although AST levels were significantly different between the BRVO group and the CRVO group, the proportion with AST levels that were abnormal was the same in each type of RVO.

In the Beaver Dam Eye Study, the prevalence and incidence of RVO were similar in men and women; however, there was a trend for female predominance, with an RVO incidence and prevalence of 55.7% and 56.4%, respectively, in women [1]. In the Korean population, the incidence of RVO is 1.28 times higher in women than in men, with the female-to-male ratio for the RVO incidence showing a rapid increase after middle age [17]. Park et al suggested that this change could be closely related to menopausal transition, although they did not perform detailed analyses according to the type of RVO [17]. In the current study, the BRVO group showed female predominance (55%), as observed in previous studies [1, 17], while the CRVO group showed slight male predominance (52%). Although the reason for the latter finding is not clear, we speculate that systemic conditions other than sex may have influenced CRVO development. Genetic and environmental differences between men and women may also have influenced the results.

As observed in previous studies, hypertension was the most common underlying disease in RVO patients in the present study, followed by DM, gastric ulcer, dyslipidemia, and stroke. A strong relationship between RVO and cardiovascular diseases has been reported over the years. Many researchers have found a significantly higher prevalence of hypertension in RVO patients [18–20], along with an increased risk of stroke, atrial fibrillation, and acute myocardial infarction after RVO occurrence [21–23]. A recent meta-analysis of published studies suggested that 48% of RVO cases can be attributed to hypertension, 20% to hyperlipidemia, and 5% to diabetes [24]. Of note, Hayreh et al reported that a higher prevalence of hypertension was observed in BRVO patients than in CRVO and hemi-CRVO patients [8]. They suggested that arteriosclerotic changes in the retinal arteries may play a role in the development of BRVO, but not CRVO, and there was no detailed analysis with SBP and DBP [8]. In the present study, the prevalence of hypertension was similar in both groups, although patients with BRVO showed significantly higher DBP (by 7 mmHg) than did those with CRVO. With this finding, we could assume that atherosclerotic change caused by underlying hypertension in the artery might result in a narrowing of the neighboring venous lumen in both BRVO and CRVO and diastolic arterial hypertension might influence to a greater extent the arteriovenous crossing than the lamina cribrosa where CRVO occurs; however, the role of hypertension in the pathogenesis of CRVO remains to be elucidated [15].

The prevalence of DM and the blood glucose level were significantly higher in patients with CRVO than in those with BRVO in the present study. While some previous studies on RVO did not find a difference in the prevalence of DM between CRVO and BRVO, they found a difference between ischemic and non-ischemic CRVO [8]. The multicenter Eye Disease Case-Control Study Group reported an increased risk of arterial hypertension and cardiovascular disease in BRVO and CRVO patients, with the latter also showing a high risk of DM [18, 19]. These results favor the association of DM with RVO in the present study. Although we could not elucidate the exact cause, the vascular changes in diabetic patients may influence CRVO development. Further studies may be necessary.

The CKD prevalence was higher in the CRVO group than in the BRVO group in the present study. In a previous study, chronic renal failure was observed in 4.9% of patients with ischemic CRVO and in 1.6% of patients with BRVO [8]; these findings were very similar to ours (4.3% in CRVO and 1.8% in BRVO). Another study also claimed that the prevalence of chronic renal failure was significantly higher in CRVO patients than in BRVO patients (15.6% vs. 11.9%; *P* < .0001) [23]. The role of CKD in RVO pathogenesis has not been widely reported; the influence of CKD on retinal vessels was suggested in the setting of diabetic retinopathy [25, 26]. It has been postulated that circulating toxins and excitotoxic metabolites in the setting of renal insufficiency cause retinal microvascular damage in diabetic retinopathy. Oxidative stress and elevated nitric oxide levels in the blood stream catalyze endothelial and neuronal damage, which has been noted in cardiorenal syndromes. Since there was significantly higher prevalence of abnormal serum BUN and creatinine levels in the CRVO than in the BRVO group, circulating toxins observed in CKD patients could have caused persistent endothelial damage and could have resulted in CRVO development. Alternatively, arterial hypertension resulted from CKD might play a role in the development of RVO. Whether either, both, or none of the mechanisms suggested above caused the development of RVO, the correlation of CKD with the development of RVO warrants further study.

An interesting finding in the present study is the elevated serum homocysteine level in the CRVO group. Elevated plasma homocysteine is a known risk factor for arterial and venous thrombosis [27]. The mechanisms by which homocysteine damages the blood vessel wall seem to be multifactorial [28, 29]. With regard to the pathogenesis of RVO, the role of homocysteine remains controversial. Some studies reported significantly higher plasma homocysteine levels in RVO [30–32], while others have failed to reveal any relationship [33, 34]. These discrepancies in findings may be due to the small sample sizes in many of the studies, heterogeneous subject populations, and variations in analysis methods. In this regard, the present study overcomes the limitations of previous studies by including a sufficient number of patients belonging to a single ethnicity and by using identical analysis methods.

PT is a measure of the integrity of the extrinsic and final common pathways of the coagulation cascade, which comprises the tissue factor and factors VII, II, V, and X and fibrinogen. A shorter PT in CRVO than in BRVO may be related to a higher serum homocysteine level in the CRVO patients in our study. Elevated homocysteine levels cause thrombosis via several mechanisms, such as increased tissue factor expression, attenuated anticoagulant processes, enhanced platelet activity, increased thrombin generation, augmented factor V activity, impaired fibrinolytic potential, and vascular injury [35]. The increased serum homocysteine level may have affected tissue factor and factor V expression, thus resulting in a decreased PT.

NLR is an inflammatory marker for diseases related to thrombosis and inflammation. Some studies have shown that high neutrophil levels are associated with a poor prognosis and increased mortality in patients with cardiovascular disease [36–38]. Dursun et al explored the association between NLR and RVO development and found a higher neutrophil count in RVO patients than in normal controls [39]. In the present study, the neutrophil and lymphocyte proportions were higher and lower, respectively, in the CRVO group than in the BRVO group. However, we failed to find significant differences in NLR between the two groups, although there was a trend of increased neutrophils and decreased lymphocytes in the CRVO group compared to the BRVO group.

The serum AST level was significantly different between the two groups in the present study. AST is measured with a liver function test, and higher readings may suggest inflammation of liver cells or the death of some cells due to liver damage. Nevertheless, the mean values were within the normal range in both groups. It may be controversial to use AST as a distinguishing risk factor for BRVO, considering that serum AST levels could be affected by several conditions such as alcohol consumption, certain medications, and hyperlipidemia.

The present study has some limitations. The first and most important limitation is that we could not evaluate the severity of RVO because electrical medical chart or fluorescence angiography findings are not included in the CDW database. Hayreh et al reported systemic diseases associated with six different types of RVO [8]. RVO could be divided into different entities according to the extent of ischemia or involved area, and each RVO subgroup may exhibit different clinical characteristics. However, the extensive subgrouping, which resulted in few subjects per subgroup, could have affected the statistical results. Larger samples with simple grouping and analysis accompanied by laboratory findings could overcome this limitation. Second, it is possible that some patients who had been examined within a different medical system prior to visiting our hospital could have a previous diagnosis of RVO. However, we believe that there were few such cases and did not affect the results. Third, longitudinal analysis with changes in laboratory data or systemic conditions after RVO development and recovery were not evaluated. Nevertheless, to the best of our knowledge, this is thus far one of the largest studies analyzing laboratory data for RVO patients. All patients belonged to a single ethnicity, which minimized genetic differences among the various risk factors. Use of the same analysis method with a single database system (CDW system of HUMC) is another strength of our study.

Our findings suggest that BRVO and CRVO exhibit different systemic conditions at the time of diagnosis. While BRVO may be more common in women, CRVO may be associated with a higher prevalence of DM and CKD as well as increased homocysteine levels and higher prevalence of abnormal serum BUN and creatinine levels. Detailed investigation of risk factors and the severity of RVO, as well as the assessment of changes in laboratory findings during the clinical course, could provide insight into the pathophysiology of RVO and assist in RVO prevention.

## Supporting information

S1 File The demographics and clinical data of the subjects. (XLSX)

S1 Table Diagnostic criteria for systemic comorbidities. (DOCX)
