# Supplementary material for: Comparison of systemic conditions at diagnosis between central retinal vein occlusion and branch retinal vein occlusion
Source: PLoS One. 2019 Aug 8;14(8):e0220880. doi: 10.1371/journal.pone.0220880 (PMC6687171; doi:10.1371/journal.pone.0220880)
Supplement: S1 Table — (DOCX) [file pone.0220880.s002.docx]

**Supplementary Table 1. Diagnostic criteria for systemic comorbidities**

| **Systemic comorbidity** | **Korean Standard Classification of Diseases code** |
| --- | --- |
| **Hypertension** | I10, I11, I12, I13, I14, I15 |
| **Diabetes mellitus** | E10, E11, E12, E13, E14 |
| **Ischemic heart disease** | I20, I21, I22, I23, I24, I25 |
| **Dyslipidemia** | E78 |
| **Cerebral infarction** | I63 |
| **Cerebral hemorrhage** | I60, I61, I62 |
| **Arrhythmia** | I44, I45, I46, I47, I48, I49 |
| **Chronic kidney disease** | N18 |
| **Gastroduodenal ulcer or inflammation** | K25, K26, K27, K29 |
| **Benign prostatic hyperplasia** | N40 |
